# The Treatment of Aneurism

**Published:** 1895-12-28

**Authors:** A. Pearce Gould

**Affiliations:** Senior Assistant Surgeon to the Middlesex Hospital.


### The Treatment of Aneurism.

By A. Pearce Gould, F.R.C.S., Senior Assistant Surgeon to the Middlesex Hospital.

We have seen that aneurisms are the result of a disproportion between the pressure of the blood in the arteries and the strength of the arterial walls, and that the chief factor is the weakening of the arterial wall by injury or disease. The "rational" treatment of aneurism would, therefore, consist in a correction of this disproportion, and especially by means that would increase the power of the arterial walls to resist the pressure of the blood. Unfortunately, this is just what we are unable to do. We can lessen the blood-pressure, but we cannot re-form a disintegrated arterial wall, and the tissue which is formed in the repair of a wound in an artery, or by the organisation of a blood-clot, is deficient in just the one all-important character—the power of resisting the expansile force of the blood. From this it follows that the field of usefulness of the "medical" treatment of aneurism is limited, and that the "surgical" treatment is only really satisfactory when it succeeds in obliterating, not only the aneurism itself, but also that part of the artery from which it springs.

But if we cannot regard the medical treatment of aneurism as very efficient as a means of cure, we must not at once put it aside as useless, or even unimportant. It is not so. Many cases of aneurism—and those the most important—are not well adapted for the more reliable surgical methods of treatment, *e.g.*, aortic and intracranial aneurisms. In these, therefore, we are practically tied up to "medical" measures, and it will be well at the outset to realise just what diet and other medicinal treatment is capable of accomplishing. This is mainly the reduction of the arterial blood-pressure. We can accomplish this by a spare and dry diet, which acts by reducing the total quantity of the blood, and by securing not only local but general rest, in order to lessen the frequency and the force of the heart's contraction. Where the effect must be obtained at once, a free blood-letting should be employed. By these means—absolute rest, spare diet, and in some cases also blood-letting—the frequency and the force of the heart's action can be considerably diminished and the blood-pressure proportionately reduced. As a result we get less pain in the aneurism, a slowed, or even arrested, growth of the tumour, escape from the danger of rupture of the sac, and in some cases the improvement thus begun goes on even to complete obliteration of the sac, along with consolidation of the artery above and below the sac.

But "medical" treatment has aimed at still more. The natural process of cure of an aneurism is by the deposit of fibrin or blood-clot in the sac of the aneurism and the extension of this clot into the artery; such a clot is then organised into a firm fibrous cicatrix. It has been suggested that by diet the composition of the blood can be so altered as to increase its coagulability, and so hasten the separation of fibrin from the circulating blood. In considering this point it is important to bear in mind that the phrase "coagulability of the blood" connotes two quite distinct ideas: first, the amount of fibrin in the blood; and second, the readiness with which the fibrin coagulates. We have to admit that we are not able by diet to alter at all the tendency to or the rapidity of the coagulation of the fibrin in the circulating blood. By diet we may succeed in increasing the relative amount of fibrin in the blood, and even the absolute amount; but this is really of small moment, for the cure of an aneurism never fails for lack of fibrin in the blood, or because it is present in too small proportion. For these reasons we cannot regard medical treatment to "increase the coagulability of the blood" as of real practical value, and we believe that its utility has often been greatly overestimated.

Much has been written about the efficacy of various drugs in cases of aneurism. Of these the most valuable is opium. As an anodyne it has no rival, and in this capacity is used with the greatest advantage. It also quiets an excited heart, and is a valuable adjuvant to rest and diet in reducing arterial tension. Moreover, it soothes a restless patient, lessens his appetite, and enables him to submit cheerfully to an otherwise severe and trying regimen. In many cases, therefore, it plays a very important part, and its usefulness is great and undoubted. Iodide of potassium in large doses—as much as a drachm and a half in twenty-four hours—has been greatly vaunted, not only as a cardiac depressant, but as having a specific effect upon the nutrition of the arterial wall itself. Opinion is much divided as to its value, some authorities holding that it is of great use, and especially in the subjects of syphilitic arteritis, while others believe that any good results which have followed its administration are to be explained by its depressing influence upon the heart and the consequent diminution of the arterial tension. Direct cardiac depressants, such as aconite and veratria, have been occasionally employed as adjuvants to other means for reducing blood-pressure.

There can be little doubt that the most potent of all the means we have mentioned is rest; next to this we would place an appropriate diet, and next to that in value, and not much inferior to it, is opium, while blood-letting in appropriate cases is also very useful. It is by the diminution of the arterial tension it effects that this treatment succeeds, and the physician must judge of the hopefulness of his efforts by the obvious effects he can produce upon the pulse; this, and not any obscure change in the composition of the blood, should be the aim of the medical treatment of aneurism.

The rest that is to be enjoined is of the most absolute kind. The patient should not be allowed to move in bed for any purpose; he must not feed himself, or turn about from side to side; and some have even forbidden him to use his handkerchief. A nurse must be at hand to help him in everything. Equally important is the avoidance of all emotion; he must not transact business, engage in arguments, or expose himself to excitement of any kind.

The diet should be gradually—not suddenly—reduced to the minimum consistent with health. In particular, the fluid should be cut down to ten or even six ounces of milk or water in the twenty-four hours; and the solids may be reduced to six ounces of meat, and two to four ounces of bread with a little butter. Many patients find this *régime* very trying, and great difficulty may be found in carrying it out. This medical treatment should be well and carefully tried in all inoperable internal aneurisms, and in a modified form it should be tried before resorting to those surgical procedures which will form the subject of our next article.

